# Longitudinal Changes in Illness Acceptance, Psychological Adjustment, and Quality of Life After Robot-Assisted Radical Prostatectomy

**DOI:** 10.3390/cancers18172774

**Published:** 2026-08-26

**Authors:** Adrianna Królikowska, Kamila Rachubińska, Mariusz Panczyk, Marzena Mikła, Anna Maria Cybulska, Marta Nowak, Elżbieta Grochans, Daria Schneider-Matyka

**Affiliations:** 1 Department of Nursing, Pomeranian Medical University in Szczecin, 70-204 Szczecin, Poland; adrianna.krolikowska@pum.edu.pl (A.K.); anna.cybulska@pum.edu.pl (A.M.C.); marta.nowak@pum.edu.pl (M.N.); elzbieta.grochans@pum.edu.pl (E.G.); daria.schneider.matyka@pum.edu.pl (D.S.-M.); 2 Department of Education and Research in Health Sciences, Medical University of Warsaw, 02-091 Warsaw, Poland; mariusz.panczyk@wum.edu.pl; 3 Faculty of Nursing, University of Murcia, 30100 Murcia, Spain; marzena.mikla@um.es; 4 Murcian Institute for Biomedical Research Pascual Parrilla (IMIB), 30120 Murcia, Spain

**Keywords:** acceptance of illness, quality of life, RARP

## Abstract

**Background**: Prostate cancer is one of the most commonly diagnosed cancers in men. Robot-assisted radical prostatectomy (RARP) is one of the recommended surgical treatment options; however, knowledge regarding changes in illness acceptance, psychological adjustment, and quality of life after surgery remains limited. **Objective**: To evaluate changes in illness acceptance, psychological adjustment, and quality of life in patients undergoing RARP. **Materials and Methods**: A total of 150 patients diagnosed with prostate cancer and scheduled for RARP were enrolled. Longitudinal analysis was performed in 93 patients who completed both study assessments. Evaluations were conducted before surgery and 3–4 months postoperatively using a self-developed questionnaire, the Acceptance of Illness Scale (AIS), the Mini-Mental Adjustment to Cancer (Mini-MAC), the European Organisation for Research and Treatment of Cancer Quality of Life Questionnaire Core 30 (EORTC QLQ-C30), and the prostate cancer-specific module (EORTC QLQ-PR25). **Results**: Following RARP, illness acceptance did not change significantly after Holm adjustment (*p*_Holm_ = 0.465). Psychological adjustment showed an adverse postoperative pattern, characterised by increased anxious preoccupation, helplessness–hopelessness, and destructive style, together with reduced fighting spirit and constructive style. After adjustment across 25 paired outcome comparisons, significant increases remained in emotional and cognitive functioning scores, while constipation and diarrhoea decreased significantly. Sexual activity also decreased significantly after surgery. Among participants who completed both assessments, incontinence-aid use increased from 15/93 (16.1%) before surgery to 38/93 (40.9%) at follow-up (exact two-sided McNemar test, *p* < 0.001). **Conclusions**: During the early postoperative period following RARP, favourable changes were observed in selected quality-of-life scores, whereas illness acceptance did not change significantly after adjustment for multiple comparisons. At the same time, psychological adjustment showed an adverse pattern, characterised by reduced fighting spirit and constructive style together with increased anxious preoccupation, helplessness–hopelessness, and destructive style. Decreased sexual activity and increased incontinence-aid use further illustrate the multidimensional nature of early postoperative recovery. These exploratory findings indicate that favourable changes in selected quality-of-life outcomes do not necessarily coincide with improved psychological adaptation and support the inclusion of psycho-oncological assessment and support in postoperative care.

## Simple Summary

Prostate cancer is one of the most commonly diagnosed cancers in men. Robot-assisted radical prostatectomy (RARP) is a widely used treatment option for localized disease. Cancer treatment affects not only physical health but also psychological well-being and quality of life. However, relatively little is known about changes in illness acceptance and psychological adjustment during the early postoperative period following RARP. The aim of this study was to evaluate changes in illness acceptance, psychological adjustment, and quality of life before and 3–4 months after surgery. The results showed that illness acceptance did not change significantly after adjustment for multiple comparisons. Significant improvements were observed in emotional and cognitive functioning, accompanied by reductions in constipation and diarrhoea. At the same time, psychological adjustment showed an adverse pattern, including increased anxious preoccupation and helplessness–hopelessness, together with reduced fighting spirit and constructive style. Sexual activity also decreased significantly after surgery. In addition, the proportion of patients reporting incontinence-aid use increased during follow-up. These findings indicate that improvements in selected aspects of quality of life are not necessarily accompanied by parallel improvements in psychological adaptation. Therefore, postoperative care should address not only oncological and functional outcomes but also psychological adaptation and supportive care needs to promote recovery and improve overall quality of life.

## 1. Introduction

Prostate cancer is one of the most common cancers among men worldwide. It currently poses a significant public health problem worldwide. Prostate cancer is the second most frequently diagnosed cancer and one of the leading causes of cancer-related death among men worldwide. According to GLOBOCAN 2022, more than 1.4 million new cases and approximately 397,000 deaths were reported globally each year. In Poland, prostate cancer is currently the most frequently diagnosed malignancy among men, and its incidence has been steadily increasing. The individualisation of treatment for patients diagnosed with prostate cancer enables the optimal selection of a therapeutic approach. One of the recommended surgical treatment methods is radical prostatectomy performed using a robotic system (RARP, Robot-Assisted Radical Prostatectomy), which allows for the precise removal of the prostate gland together with the seminal vesicles and, where justified, the regional lymph nodes as well [1].

The quality of life of patients with prostate cancer is influenced by several factors, including age, disease stage, and treatment-related complications, particularly urinary incontinence and sexual dysfunction, which may significantly affect physical, psychological, and social functioning.

The process of treating a malignant tumour has a significant impact on a patient’s life, affecting physical, psychological, social and economic aspects alike. Consequently, a key element in coping with the disease is its acceptance, which is a complex, multi-stage process and, at the same time, a prerequisite for effective adaptation to the disease. Individuals with a more optimistic outlook are more likely to adopt active coping strategies and demonstrate better psychological adjustment, which may facilitate adaptation to illness and support patients’ active involvement in the treatment process [2].

Psychological adaptation to cancer is an individual process aimed at learning and, ultimately, developing mechanisms to cope with emotions and the new reality associated with the diagnosis. According to the Mini-MAC framework, psychological adjustment may be described in terms of constructive and destructive styles. The destructive style is characterised by helplessness–hopelessness and anxious preoccupation, reflecting a passive approach, psychological distress, and difficulties in adapting to the disease. In contrast, the constructive style comprises fighting spirit and positive re-evaluation and reflects active engagement, hope for recovery, and efforts to maintain control over the disease situation [3,4,5,6].

Both illness acceptance and appropriate psychological adaptation to the disease play a key role in patients’ subjective assessment of their quality of life. Understanding changes in illness acceptance, psychological adjustment and quality of life in patients treated with robotic prostatectomy may contribute to the development of more effective strategies for emotional support. Despite the growing number of studies on quality of life following robotic prostatectomy, knowledge regarding changes in illness acceptance and psychological adjustment in the early postoperative period remains limited. In particular, few longitudinal studies have simultaneously evaluated illness acceptance, psychological adjustment and quality of life in patients undergoing robotic radical prostatectomy.

The aim of this exploratory longitudinal study was to assess changes in illness acceptance, psychological adjustment to cancer, and health-related quality of life between the preoperative assessment and the assessment performed 3–4 months after RARP. Because no single confirmatory primary endpoint was prospectively specified, the outcomes were analysed as a multidimensional set of exploratory endpoints.

## 2. Materials and Methods

### 2.1. Study and Participant Characteristics

The study initially involved 150 patients diagnosed with prostate cancer who underwent surgical treatment by means of robotic radical prostatectomy. In the second phase of the study, some participants were lost to follow-up. Of the 150 patients assessed at baseline, 93 completed the follow-up assessment and were eligible for longitudinal analyses. Because some individual outcomes contained missing or not-applicable data, analyses were based on available observations and available complete pre–post pairs for each outcome. No imputation was performed. The loss of 57 participants was mainly due to a failure to respond to a repeat invitation to take part in the second phase of the study. The participants did not formally withdraw from the study; however, despite being re-invited, they did not complete the full set of questionnaires, which made it impossible to include their data in the final analysis. Consequently, only patients who completed the questionnaires both before the procedure and 3–4 months after the operation were included in the longitudinal analysis.

The study was conducted at the Department of Urology and Urological Oncology at University Clinical Hospital No. 2 in Szczecin. Patient recruitment and surgical treatment were conducted throughout 2025, and follow-up assessments were completed by the end of the same year for patients who had reached the planned 3–4-month postoperative follow-up. All study participants underwent prostatectomy using the da Vinci Surgical System (Intuitive Surgical, Inc., Sunnyvale, CA, USA).

Prior to the commencement of the study, approval was obtained from the Bioethics Committee of the Pomeranian Medical University in Szczecin on 20 November 2023 (KB.006.122.2023). The study was conducted in accordance with the principles of the Declaration of Helsinki. Participation in the study was voluntary and confidential, and participants were informed of their right to withdraw from the study at any stage.

Longitudinal studies were conducted to compare the levels of cancer acceptance, psychological adjustment and quality of life among patients before and after robotic radical prostatectomy. The study was carried out at two time points. No formal a priori sample-size calculation was performed because the study was exploratory in nature. The sample size was determined by the feasibility of recruitment during the predefined study period. A total of 150 patients were enrolled at baseline, of whom 93 completed both assessments and were included in the longitudinal analyses.

### 2.2. Data Collection Method

The first stage of the study was carried out in a hospital setting, during the patients’ hospital stay, immediately prior to the planned surgical procedure. The questionnaires were completed on the ward in the presence of a member of the research team, who provided organisational support where necessary.

The second stage of the study was carried out approximately 3–4 months after the prostatectomy. Patients were asked to complete the same set of questionnaires again. Data collection was carried out using various methods of contact, tailored to the respondents’ preferences, including by post and electronically. Of the 150 patients assessed at baseline, 93 completed the follow-up assessment and were eligible for longitudinal analyses. Individual comparisons were based on available complete pre–post pairs for each outcome. Of the 93 patients who completed the second assessment, 62 completed the questionnaires during a follow-up visit to the urology outpatient clinic, 24 completed them electronically using an online questionnaire, and 7 returned the completed paper questionnaires by post. The questionnaires were made available in both paper and electronic formats. Paper forms were handed to patients during follow-up visits at the urology clinic or when they collected their histopathological results, whilst the electronic version was sent by email in the form of a link to an online survey. Where personal contact was not possible, questionnaires were sent by post, accompanied by a return envelope. All forms of data collection were carried out in accordance with confidentiality principles and in compliance with applicable data protection regulations. Despite the use of various contact strategies, it was not possible to obtain data from all participants originally eligible for the study. Some patients did not respond to the repeat invitation to participate in the second stage of the study. The low response rate was mainly due to difficulties in establishing contact. Not all patients attended follow-up appointments at the urology clinic, nor did everyone collect their histopathological results in person. Consequently, participants who returned follow-up questionnaires were included in the longitudinal cohort, and each outcome was analysed using the available complete pre–post pairs for that specific outcome. No imputation was performed.

### 2.3. Inclusion and Exclusion Criteria

The study included patients with a confirmed diagnosis of prostate cancer who were eligible for surgical treatment in the form of robotic prostatectomy, in accordance with the guidelines of the European Association of Urology and the Polish Association of Urology. A prerequisite for participation in the study was preserved mental capacity enabling patients to complete the study questionnaires independently and with full understanding, and to give written, informed consent to participate in the research.

Patients who did not give informed consent to participate in the study were excluded, as were those with diagnosed neurodegenerative disorders, such as Alzheimer’s disease, dementia syndromes or other medical conditions preventing them from completing the questionnaires independently and with full understanding. Exclusion criteria also included severe mental disorders and clinical conditions that could pose a threat to the patient’s health or life.

### 2.4. Methods Used

Two key methods were used in the study: analysis of patients’ medical records and a diagnostic survey, employing a questionnaire technique using a self-designed questionnaire and standardised research tools. Consent and a licence to use the research tools in this study were obtained. The self-developed questionnaire consisted of 13 items and was used to collect sociodemographic, health-related, and lifestyle information. It included questions on age, education, place of residence, marital status, occupational status, self-rated general health, time since prostate cancer diagnosis, family history of cancer, financial status, physical activity, smoking, alcohol consumption, and participation in preventive examinations. The questionnaire was used solely for descriptive characterization of the study population and was not intended as a psychometric measurement instrument.

The Acceptance of Illness Scale (AIS), developed by Barbara J. Felton, Tracey A. Revenson and Gregory A. Hinrichsen, was used in a Polish adaptation and validation carried out by Zygfryd Juczyński, and is used to measure the extent to which a patient has come to terms with their cancer. The scale comprises 8 statements relating to the negative consequences of poor health. Responses are rated on a 5-point scale, which allows for a subjective assessment of the level of acceptance. Scores range from 8 to 40 points. Higher scores suggest greater acceptance of the illness, whilst lower scores indicate a lower degree of acceptance of the illness. The AIS scale, as adapted by Z. Juczyński, is characterised by satisfactory internal consistency, measured by a Cronbach’s alpha coefficient of 0.85 [7].

The Mini-MAC (Mini-Mental Adjustment to Cancer Scale), developed by Watson and colleagues, is a standardized instrument used to assess psychological adjustment to cancer. The questionnaire consists of 29 items describing patients’ responses to cancer and enables the assessment of four adjustment strategies: anxious preoccupation, fighting spirit, helplessness–hopelessness, and positive re-evaluation. Higher scores indicate greater intensity of behaviours characteristic of a given adjustment strategy. In addition, the Mini-MAC provides scores for two broader adjustment styles:

- constructive style (fighting spirit and positive re-evaluation),
- destructive style (anxious preoccupation and helplessness–hopelessness).

The Polish adaptation of the Mini-MAC demonstrated high reliability, with Cronbach’s alpha coefficients ranging from 0.87 to 0.92 for the individual subscales [8].

The EORTC QLQ-C30 questionnaire (European Organisation for Research and Treatment of Cancer Quality of Life Questionnaire—Core 30) in the Polish version developed by the European Organisation for Research and Treatment of Cancer, which is a tool used to assess the quality of life of cancer patients [9]. The questionnaire consists of 30 items assessing the patient’s functioning, the severity of disease symptoms and general health. Validation studies have shown that the questionnaire’s scales achieve Cronbach’s alpha values above 0.70, indicating good reliability and internal consistency [10].

The EORTC QLQ-C30 and EORTC QLQ-PR25 questionnaires were administered and scored in accordance with the official EORTC Scoring Manual. The Polish language versions of both questionnaires have previously undergone psychometric validation. According to the published validation study of the Polish versions [11], the questionnaires demonstrated good reliability, with Cronbach’s alpha coefficients ranging from 0.849 to 0.908. As the present study was not designed as a psychometric validation study, no additional assessment of internal consistency was performed. The incontinence aid and sexual functioning domains are conditional and are scored only for patients for whom the respective items are applicable. Consequently, the number of available observations for these domains was lower than for the other QLQ-PR25 domains.

### 2.5. Statistical Analysis

Quantitative variables are presented as means and standard deviations or medians and interquartile ranges, as appropriate, whereas categorical variables are presented as frequencies and percentages. Paired preoperative and postoperative measurements were compared using the Wilcoxon signed-rank test. Two-sided asymptotic *p*-values were derived from the standardized, tie-adjusted Wilcoxon *Z* statistic without continuity correction. Zero pre-post differences were excluded from ranking, whereas tied non-zero absolute differences were assigned average ranks. Effect size *r* was calculated as |*Z*|/√*N*, where *N* denotes the number of complete paired observations; zero-difference pairs were retained in the denominator.

To assess potential attrition-related selection bias, baseline AIS, Mini-MAC, EORTC QLQ-C30, and EORTC QLQ-PR25 scores were compared between participants who completed the postoperative assessment and those lost to follow-up. Standardized mean differences (SMDs) and exploratory Mann–Whitney U tests were calculated. These analyses were exploratory and were not included in the family of comparisons subjected to Holm adjustment.

The incontinence-aid and sexual-functioning domains of the EORTC QLQ-PR25 are conditional. Therefore, domain applicability was analysed separately from the corresponding conditional scores. Paired transitions between assessments were summarized in 2 × 2 tables and evaluated using exact two-sided McNemar tests. The conditional scores were summarized descriptively and were not included in the multiplicity-adjusted family of outcome comparisons.

No single confirmatory primary endpoint was prospectively specified; therefore, all inferential analyses were considered exploratory. To control the family-wise type I error rate arising from multiple testing, the Holm procedure was applied to a defined family of 25 paired outcome comparisons comprising the AIS, Mini-MAC, EORTC QLQ-C30, and EORTC QLQ-PR25 outcomes. Holm-adjusted *p*-values were calculated from the full-precision unadjusted *p*-values. Statistical significance was defined as a two-sided Holm-adjusted *p*-value < 0.05.

Descriptive analyses were performed using jamovi version 2.6.44. The final Wilcoxon signed-rank analyses, effect-size calculations, Mann–Whitney U tests, exact McNemar tests, internal consistency checks, and Holm adjustment were performed in Python version 3.13.15 using NumPy version 2.1.3, pandas version 2.2.3, SciPy version 1.16.3, and statsmodels version 0.14.6.

## 3. Results

### 3.1. Characteristics of the Study Group

A total of 150 men with a diagnosis of prostate cancer who were eligible for robot-assisted radical prostatectomy were recruited into the study. The longitudinal cohort comprised 93 patients who completed both study assessments; outcome-specific numbers of complete pairs varied because of missing or not-applicable data. During follow-up, 57 participants were lost to follow-up, corresponding to an attrition rate of 38%. Baseline characteristics of the 93 participants who completed follow-up and the 57 participants who did not are presented in Supplementary Table S1. The largest baseline difference was observed for the conditional sexual-functioning score (SMD = −0.35), followed by cognitive functioning (81.34 vs. 85.96 points; SMD = −0.28), indicating higher baseline scores among participants lost to follow-up. All remaining standardized mean differences were ≤0.23.

In the group of 150 patients, the mean age of the participants was 68.3. In the analysed group, the most numerous subgroups were patients with secondary education—36 per cent, those living in towns and cities with a population of over 100,000—32.7 per cent, those in formal relationships—82 per cent, and pensioners—56.7 per cent. Over half of the respondents rated their financial situation as ‘rather good’—52 per cent.

Clinical and oncological data were available for all 150 patients. The mean preoperative PSA level was 9.51 ng/mL (SD = 5.98), and the median PI-RADS score was 4.00 (IQR = 1.00). The median Gleason score was 7.00 (IQR = 1.00), indicating that most patients had tumours of intermediate histological grade. The median ISUP grade was 2.00 (IQR = 2.00), with the majority of patients classified as ISUP grade 1 or 2. According to the Briganti nomogram, 51.3% of patients were classified as having a low risk of lymph node metastases, defined as a Briganti score < 5%. Detailed clinical and oncological characteristics are presented in Supplementary Table S4. Postoperative complications occurred in 13 of 150 patients (8.7%) (Supplementary Table S5). The recorded complications included anemia (*n* = 4), lymphocele (*n* = 2), lymphocele with elevated inflammatory markers (*n* = 1), serous wound drainage (*n* = 1), anastomotic leakage (*n* = 1), severe abdominal pain (*n* = 1), urinary leakage (*n* = 1), post-anesthetic organic delirium (*n* = 1), and bladder bleeding (*n* = 1).

### 3.2. Assessment of Illness Acceptance, Psychological Adjustment and Quality of Life in Patients Prior to RARP

In the group of patients assessed prior to robotic prostatectomy, the mean illness acceptance score on the AIS was 32.41 points (SD = 7.47), indicating a relatively high level of illness acceptance. With regard to psychological adjustment to cancer assessed using the Mini-MAC, high and very high levels of the constructive style predominated, accounting for 52.0% of respondents, whereas a low level was observed in 3.3% and no very low scores were recorded. For the destructive style, very low scores were most common (63.1%), followed by low scores (28.9%); average and high levels were observed in 6.7% and 1.3% of patients, respectively, and no very high scores were recorded.

Among the individual Mini-MAC adjustment strategies, the highest mean scores were observed for fighting spirit (M = 23.45, SD = 3.28) and positive re-evaluation (M = 21.35, SD = 3.22), whereas lower scores were recorded for anxious preoccupation (M = 13.72, SD = 4.29) and helplessness–hopelessness (M = 10.69, SD = 3.69). The mean constructive style score was 44.79 (SD = 5.57), compared with 24.43 (SD = 7.28) for the destructive style, indicating an overall predominance of the constructive style.

Quality of life was assessed using the EORTC QLQ-C30 questionnaire, which comprises functional scales, symptom scales, and a global health status/quality-of-life scale. Participants reported relatively high levels of physical functioning (M = 82.52, SD = 15.26), role functioning (M = 87.58, SD = 19.96), cognitive functioning (M = 83.11, SD = 16.78), and social functioning (M = 84.12, SD = 18.00). Emotional functioning was also relatively high (M = 75.69, SD = 16.63). Among the symptom scales, the highest mean scores were observed for constipation (M = 32.89, SD = 22.92), followed by diarrhoea (M = 23.71, SD = 20.61), pain (M = 20.44, SD = 17.07), and fatigue (M = 18.81, SD = 16.11). The lowest mean scores were recorded for nausea and vomiting (M = 2.68, SD = 8.00) and dyspnoea (M = 6.04, SD = 15.53). The mean global health status/quality-of-life score was 74.11 (SD = 20.85), indicating a generally favourable self-assessment of overall quality of life.

The EORTC QLQ-PR25 module was used to assess prostate cancer-specific quality-of-life domains. The highest mean symptom scores were observed for the incontinence-aid domain (M = 20.99, SD = 33.52) and urinary symptoms (M = 20.15, SD = 15.35). Bowel symptoms were infrequent and of low severity (M = 5.71, SD = 8.39), whereas hormonal symptoms had a mean score of 12.68 (SD = 9.66). Regarding sexual health, the mean sexual activity score was 48.82 (SD = 25.73), while sexual functioning among respondents with available data was relatively high (M = 76.53, SD = 18.85) (Table 1).

### 3.3. Assessment of Illness Acceptance, Psychological Adjustment and Quality of Life in Patients Following RARP

Among the 93 patients assessed 3–4 months after robot-assisted radical prostatectomy, the mean AIS score was 35.08 (SD = 5.82), indicating a relatively high level of illness acceptance.

Assessment of psychological adjustment using the Mini-MAC showed a predominance of the constructive style, with a mean constructive style score of 41.46 (SD = 4.58), compared with 27.84 (SD = 6.55) for the destructive style. Among the individual adjustment strategies, the mean scores were 20.92 (SD = 3.12) for fighting spirit, 20.54 (SD = 2.74) for positive re-evaluation, 15.27 (SD = 3.62) for anxious preoccupation, and 12.57 (SD = 3.33) for helplessness–hopelessness.

Analysis of the EORTC QLQ-C30 showed high postoperative scores for physical functioning (M = 87.67, SD = 17.91) and cognitive functioning (M = 93.73, SD = 14.73). Symptom scores were generally low, including fatigue (M = 16.49, SD = 17.84) and diarrhoea (M = 11.47, SD = 24.32). The mean global health status/quality-of-life score was 71.86 (SD = 17.93).

Within the EORTC QLQ-PR25, the highest mean symptom score was observed for the incontinence-aid domain (M = 40.35, SD = 35.65), whereas urinary symptoms had a mean score of 15.62 (SD = 16.72). Bowel symptoms (M = 5.73, SD = 13.46) and hormonal symptoms (M = 9.20, SD = 11.19) were less pronounced. The mean sexual activity score was 24.91 (SD = 26.08), while the mean sexual functioning score was 60.80 (SD = 24.90). Considerable variability was observed across several postoperative domains, particularly those related to urinary and sexual functioning.

Among the 93 patients who completed both assessments, the incontinence-aid domain was applicable to 15 patients (16.1%) at baseline and 38 patients (40.9%) at follow-up. The sexual-functioning domain was applicable to 58 patients (62.4%) at baseline and 54 patients (58.1%) at follow-up (Table 2).

### 3.4. Comparison of Acceptance, Psychological Adjustment to Cancer and Quality of Life in Patients Before and After RARP Prostatectomy

Pre- and post-surgery comparisons were performed using available complete pairs for each outcome; therefore, the number of paired observations varied across analyses (*n* = 54–93).

Following robot-assisted radical prostatectomy (RARP), illness acceptance increased numerically from a median of 34.0 to 37.0 points; however, this difference was statistically significant only before multiplicity adjustment (*p* = 0.039) and did not remain significant after Holm correction (*p*_Holm_ = 0.465).

Analysis of the Mini-MAC questionnaire demonstrated an adverse postoperative pattern of psychological adjustment. After Holm correction, anxious preoccupation increased (median: 13.0 vs. 15.0; *p*_Holm_ = 0.037), fighting spirit decreased (24.0 vs. 21.0; *p*_Holm_ < 0.001), helplessness–hopelessness increased (9.0 vs. 13.0; *p*_Holm_ < 0.001), constructive style decreased (6.0 vs. 6.0 sten points, with a significant shift in the score distribution; *p*_Holm_ < 0.001), and destructive style increased (2.0 vs. 3.0 sten points; *p*_Holm_ = 0.003). Positive re-evaluation did not change significantly (*p* = 0.162; *p*_Holm_ = 1.000).

Within the EORTC QLQ-C30, significant postoperative improvements remained after multiplicity adjustment for emotional functioning (75.0 vs. 100.0; *p*_Holm_ = 0.001) and cognitive functioning (83.3 vs. 100.0; *p*_Holm_ < 0.001). Constipation also decreased significantly (33.3 vs. 0.0; *p*_Holm_ = 0.001), and a significant reduction in diarrhoea remained after Holm correction (33.3 vs. 0.0; *p*_Holm_ = 0.011). Physical functioning improved in the unadjusted analysis (*p* = 0.008), but this difference did not remain significant after Holm correction (*p*_Holm_ = 0.123). Similarly, reductions in appetite loss (*p* = 0.018; *p*_Holm_ = 0.253) were significant only before multiplicity adjustment. No significant adjusted changes were observed in role functioning, social functioning, fatigue, nausea and vomiting, pain, dyspnoea, insomnia, or global health status/quality of life.

Within the EORTC QLQ-PR25, sexual activity decreased significantly after surgery and remained significant after Holm correction (41.7 vs. 16.7; *p*_Holm_ < 0.001). Reductions in urinary symptoms (*p* = 0.039; *p*_Holm_ = 0.465) and hormonal symptoms (*p* = 0.030; *p*_Holm_ = 0.389) were observed in the unadjusted analyses but did not remain significant after multiplicity adjustment. No significant adjusted changes were observed for Bowel symptoms.

Overall, 15 of the 25 paired comparisons were statistically significant before multiplicity adjustment, whereas 10 remained statistically significant after application of the Holm procedure. Detailed descriptive statistics, numbers of paired observations, unadjusted and Holm-adjusted *p*-values, and effect sizes are presented in Table 3.

Among follow-up completers, incontinence-aid use increased from 15/93 (16.1%) before surgery to 38/93 (40.9%) at follow-up. Thirty-one participants started using an incontinence aid, whereas eight ceased using one; the paired change was statistically significant (exact two-sided McNemar test, *p* < 0.001; Supplementary Table S2).

The applicability of the sexual-functioning domain was examined separately because this is a conditional EORTC QLQ-PR25 domain. Among the 93 participants who completed both assessments, the domain was applicable to 58 participants (62.4%) before surgery and 54 participants (58.1%) at follow-up. Transition analysis showed that 22 participants entered the subgroup for whom the domain was applicable, whereas 26 left this subgroup; 32 participants remained in the applicable subgroup at both assessments. The paired change in domain applicability was not statistically significant according to the exact two-sided McNemar test (*p* = 0.665; Supplementary Table S3).

## 4. Discussion

The observed findings should be interpreted in the context of the early postoperative period, during which functional recovery following RARP is still ongoing. The results obtained in our own research on illness acceptance, psychological adjustment and quality of life in patients following robotic prostatectomy indicate the complex nature of patients’ adaptation to cancer. This study provides information on changes in illness acceptance, psychological adjustment and quality of life in patients who have undergone robotic radical prostatectomy. An important finding was the contrasting pattern observed after surgery: illness acceptance remained statistically unchanged after adjustment for multiple comparisons, whereas psychological adjustment showed an adverse postoperative pattern.

The results indicate that the study participants demonstrated a relatively high level of illness acceptance. This may stem from the fact that patients eligible for surgical treatment perceive the procedure as a realistic chance of successful treatment and recovery, which fosters a more positive attitude towards the disease. Similar observations have also been reported by other authors, highlighting the importance of hope associated with the possibility of effective treatment in the psychological adaptation process of cancer patients [3].

Although illness acceptance scores were higher at the postoperative assessment than before surgery, this difference did not remain statistically significant after adjustment for multiple comparisons using the Holm procedure. Therefore, the observed increase should not be interpreted as a significant improvement in illness acceptance during the early postoperative period. These findings should also be considered in the context of previous research conducted at our centre in a different group of patients with prostate cancer, in which illness acceptance and psychological adjustment were assessed using the AIS and Mini-MAC instruments [12].

Regarding psychological adjustment, a constructive style predominated among patients and was driven primarily by fighting spirit and positive re-evaluation, which are considered the most adaptive adjustment strategies in the context of cancer. This may stem from the fact that, in the preoperative period, patients mobilise their psychological resources in the face of planned treatment of a potentially radical nature, which fosters adaptive adjustment strategies. Similar findings were also reported in studies by other authors, in which cancer patients most frequently exhibited a constructive style of adaptation [8].

As part of our own research, we also assessed quality of life. Patients demonstrated relatively favourable levels of quality of life, particularly in terms of physical, emotional and social functioning. Overall health was described as satisfactory. The most commonly reported complaints included constipation, diarrhoea, pain and fatigue. However, despite the presence of these symptoms, patients generally rated their overall quality of life as good. Bayked et al. presented different findings, reporting a moderate level of quality of life in a group of cancer patients undergoing chemotherapy. These differences may stem from the fact that chemotherapy is associated with more severe adverse effects and may significantly reduce patients’ subjective assessment of their well-being. It should be emphasised, however, that despite the presence of symptoms, the majority of respondents in this group also reported good general health, particularly with regard to social functioning. As in our own study, diarrhoea was among the more frequently reported symptoms and was characterised by moderate severity [13].

In the prostate cancer-specific quality-of-life analysis, the highest mean symptom scores were observed for the incontinence-aid domain and urinary symptoms, while sexual functioning among respondents with available data was relatively high. The most frequently reported problem was urinary incontinence, a finding also confirmed by studies by other authors on this patient group [14].

In terms of sexual function, the participants exhibited a moderate level of sexual activity, whilst reporting a relatively positive self-assessment of this aspect of their lives. Some reports suggest the possibility of a gradual improvement in sexual function following robotic prostatectomy, whilst other authors emphasise the persistence of erectile dysfunction. According to a study by Ward et al., the majority of patients who underwent robotic prostatectomy regained sexual function within three months. However, the results of Capogrosso’s study indicate that the vast majority of patients undergoing radical prostatectomy complain of post-operative sexual dysfunction. In Shiraishi’s studies, which assessed the sexual function of patients following robot-assisted radical prostatectomy (RARP), attention was drawn to the lack of improvement in sexual satisfaction following RARP. Erectile dysfunction, one of the most common post-operative complications, also significantly contributed to a reduction in quality of life in this group of patients [15,16,17].

Our findings are consistent with previous studies indicating that sexual health remains one of the most challenging aspects of recovery following radical prostatectomy. In our study, sexual activity decreased significantly after surgery. The applicability of the conditional sexual-functioning domain did not change significantly between assessments, with the domain being applicable to 58 participants before surgery and 54 participants at follow-up. The conditional sexual-functioning scores were available in both assessments for only 32 participants and were therefore summarized descriptively. These findings highlight the complexity of evaluating sexual health outcomes following surgical treatment.

In the analysed group of patients who had undergone surgery, a relatively high level of illness acceptance was observed, along with a predominance of the constructive style, based mainly on a ‘fighting spirit’ approach. Quality of life following treatment was assessed as relatively good; however, considerable individual variability was observed in the domains of sexual activity and sexual functioning. A discrepancy was observed between illness acceptance, which did not change significantly after adjustment for multiple comparisons, and psychological adjustment, which deteriorated following surgery. This may indicate that cognitive acceptance is not always accompanied by concurrent emotional adaptation. Reports by Xiang et al. and Anguas-Gracia et al. describe the multidimensional nature of the consequences of prostate cancer treatment [18,19].

The results of our study are consistent with the cited reports and demonstrate a similar pattern of changes in quality of life following surgical treatment for prostate cancer.

In the early postoperative period following robotic radical prostatectomy, illness acceptance scores were higher than before surgery; however, this difference did not remain statistically significant after adjustment for multiple comparisons. At the same time, psychological adjustment showed an adverse pattern, with increases in anxious preoccupation, helplessness–hopelessness, and the destructive style, together with decreases in fighting spirit and the constructive style. These findings are consistent with Ośmiałowska’s observations that cancer patients characterised by a constructive style had a higher quality of life, whereas a stronger destructive style was associated with poorer quality of life [4].

The results obtained in our study showed increases in emotional and cognitive functioning scores during the early postoperative period following robot-assisted radical prostatectomy, together with reductions in constipation and diarrhoea. Participants lost to follow-up had higher baseline cognitive-functioning scores than follow-up completers (85.96 vs. 81.34 points; SMD = −0.28). Therefore, selective retention of participants with better baseline cognitive functioning does not explain the observed postoperative increase. However, this finding should not be interpreted as evidence of a causal improvement in cognitive function attributable to RARP. The baseline assessment was performed immediately before surgery, when anticipatory stress may have temporarily affected self-reported cognitive functioning. In addition, the mode of questionnaire administration differed between assessments, which may have influenced patient-reported scores independently of true clinical change. Attrition related to unmeasured characteristics or unobserved postoperative outcomes also cannot be excluded. Our findings partially align with those reported by Karagiotis et al., who observed stable quality-of-life outcomes in the physical and emotional functioning domains among patients treated with robot-assisted prostatectomy [20].

In turn, Holze et al. demonstrated that patients who underwent RARP returned more quickly to their preoperative quality of life, particularly in terms of social functioning, compared with patients treated using the laparoscopic technique [21]. Although our study did not include a comparison between robotic and laparoscopic surgery, these studies are cited solely to provide context for the interpretation of our findings.

Analysis of our study results showed reductions in urinary and hormonal symptoms following surgical treatment; however, these changes did not remain statistically significant after adjustment for multiple comparisons. Sexual activity decreased significantly after surgery. The applicability of the conditional sexual-functioning domain did not change significantly, whereas the corresponding score was summarized descriptively because paired data were available only for a subset of participants. Similar improvements in urinary tract function after robot-assisted prostatectomy were reported by Lindenberg et al., who demonstrated more favourable urinary outcomes than those observed after the laparoscopic approach [22]. Persistent difficulties in sexual functioning after surgery and the limited effectiveness of treatment for erectile dysfunction have also been described [23]. These findings emphasize that maintaining satisfactory sexual health following prostatectomy remains a significant clinical challenge requiring comprehensive, multidisciplinary care.

An analysis of the results suggests that urinary symptoms and changes in sexual health remain important factors influencing the quality of life of patients after radical prostatectomy. Similar findings were reported by Odeo et al., who demonstrated that sexual functioning is one of the factors reducing quality of life in patients with prostate cancer [24].

These findings are consistent with recent longitudinal studies showing that urinary and sexual dysfunction remain among the most important determinants of health-related quality of life following radical prostatectomy, particularly during the first postoperative months, although gradual recovery may occur over time [25,26,27].

The value of this study lies in its simultaneous, longitudinal Assessment of illness acceptance, psychological adjustment and quality of life in a specific group of patients who have undergone robotic prostatectomy. The results obtained indicate that favourable changes in selected quality-of-life outcomes do not always translate into better psychological adjustment, which represents a significant contribution to our understanding of the process of adapting to cancer.

Taken together, these findings highlight the importance of comprehensive postoperative care that includes not only functional recovery, but also psychological assessment and support to improve patients’ overall quality of life.

### 4.1. Limitations of the Study

The study has several limitations. First, it was conducted at a single clinical centre, which may limit the generalisability of the findings. Second, the high rate of participant loss between the first and second stages of the study may have affected the representativeness of the results. Baseline characteristics of participants who completed and did not complete follow-up were compared and are presented in Supplementary Table S1. The largest baseline differences were observed for the conditional sexual-functioning score (SMD = −0.35) and cognitive functioning (SMD = −0.28), indicating higher baseline scores among participants lost to follow-up. However, participants lost to follow-up had higher, rather than lower, baseline cognitive-functioning scores, suggesting that selective retention of participants with better baseline cognitive functioning does not explain the postoperative increase observed among completers. Nevertheless, attrition bias related to unmeasured characteristics or unobserved postoperative outcomes cannot be excluded. Furthermore, follow-up questionnaires were completed using different modes of administration (during outpatient follow-up visits, online, or by post), which may have introduced response bias.

Third, baseline data were collected immediately before surgery, when patients may have experienced increased anticipatory stress. Moreover, the follow-up assessment was performed only once, at 3–4 months after RARP, representing the early postoperative period rather than the full recovery trajectory. Therefore, the observed changes should be interpreted as differences between two specific time points rather than as the long-term course of psychological adjustment and quality of life following RARP. Furthermore, the absence of a control group limits the ability to distinguish changes related to RARP from those associated with the natural course of recovery over time.

Finally, although selected clinical and oncological data were collected, detailed surgical variables, including surgeon experience, institutional surgical volume, and nerve-sparing status, were not recorded. Although some patients received androgen deprivation therapy (ADT) according to clinical indications, information regarding ADT exposure and the timing of its initiation was not systematically collected as a study variable. Therefore, the number of patients receiving ADT and the potential influence of ADT on the observed changes in psychological adjustment and sexual activity could not be reliably assessed. Consequently, the potential influence of these factors on psychological adjustment and quality of life could not be evaluated, which may limit the generalisability of the findings to other clinical settings.

An additional limitation is that no single confirmatory primary endpoint was prospectively specified. The analyses therefore have an exploratory character. Although the Holm procedure was applied to control the family-wise type I error across the defined family of 25 paired outcome comparisons, the findings should be interpreted cautiously and confirmed in studies with prospectively specified primary endpoints and an a priori sample-size calculation.

### 4.2. Practical Implications

The study’s findings indicate that relatively high illness acceptance may coexist with difficulties in psychological adaptation, which supports monitoring adaptive difficulties in the early postoperative period. Assessing quality of life in terms of sexual health and urinary symptoms also remains a key element of care for patients following radical prostatectomy. These complications can significantly affect patients’ quality of life. Patient education regarding the consequences of treatment and methods of urological rehabilitation also remains a key element of care. The results of this study may form the basis for developing support programmes for men following radical treatment for prostate cancer and for establishing standards of comprehensive post-operative care. These measures may contribute to improving the quality of life and psychological adjustment of patients following radical prostatectomy.

## 5. Conclusions

Patients who underwent robot-assisted radical prostatectomy did not demonstrate a statistically significant change in illness acceptance after adjustment for multiple comparisons. During the early postoperative period following RARP, significant increases in emotional and cognitive functioning scores were observed, together with reductions in constipation and diarrhoea, whereas the nominal improvement in physical functioning did not remain significant after Holm correction. Reductions in urinary and hormonal symptoms were observed only in the unadjusted analyses and were no longer statistically significant after multiplicity adjustment. Sexual activity decreased significantly after surgery, and the proportion of patients reporting incontinence-aid use increased during follow-up. Psychological adjustment showed an adverse postoperative pattern, characterised by increased anxious preoccupation, helplessness–hopelessness, and destructive style, together with reduced fighting spirit and constructive style. These findings indicate that favourable changes in selected quality-of-life outcomes during the early postoperative period following RARP are not necessarily accompanied by parallel improvements in psychological adaptation. The findings are exploratory and should be confirmed in prospective longitudinal studies with prespecified primary endpoints and an a priori sample-size calculation.

## Figures and Tables

**Table 1:** Baseline characteristics of the entire study cohort at the first assessment, including disease acceptance (AIS), psychological adjustment to cancer (Mini-MAC), and quality of life (EORTC QLQ-C30 and EORTC QLQ-PR25) (*n* = 150).

Instrument	Variable	*n*	M ± SD	Me	IQR	Min–Max
AIS		149	32.41 ± 7.47	34.00	9.00	8.00–40.00
Mini-MAC	Anxious preoccupation	150	13.72 ± 4.29	13.00	6.00	7.00–27.00
	Fighting spirit	150	23.45 ± 3.28	24.00	5.00	17.00–28.00
	Helplessness–hopelessness	149	10.69 ± 3.69	10.00	6.00	7.00–27.00
	Positive re-evaluation	150	21.35 ± 3.22	22.00	4.00	10.00–28.00
	Constructive style	150	44.79 ± 5.57	45.00	10.00	29.00–55.00
	Destructive style	149	24.43 ± 7.28	24.00	11.00	14.00–48.00
EORTC QLQ-C30	Physical functioning	150	82.52 ± 15.26	86.67	20.00	20.00–100.00
	Role functioning	149	87.58 ± 19.96	100.00	16.67	0.00–100.00
	Emotional functioning	149	75.69 ± 16.63	75.00	22.22	16.67–100.00
	Cognitive functioning	149	83.11 ± 16.78	83.33	33.33	16.67–100.00
	Social functioning	149	84.12 ± 18.00	83.33	33.33	33.33–100.00
	Fatigue	150	18.81 ± 16.11	22.22	19.44	0.00–88.89
	Nausea and vomiting	149	2.68 ± 8.00	0.00	0.00	0.00–33.33
	Pain	150	20.44 ± 17.07	16.67	16.67	0.00–100.00
	Dyspnoea	149	6.04 ± 15.53	0.00	0.00	0.00–100.00
	Insomnia	149	13.20 ± 19.30	0.00	33.33	0.00–66.67
	Appetite loss	149	11.19 ± 16.26	0.00	33.33	0.00–66.67
	Constipation	149	32.89 ± 22.92	33.33	0.00	0.00–100.00
	Diarrhoea	149	23.71 ± 20.61	33.33	33.33	0.00–100.00
	Global health status/QoL	149	74.11 ± 20.85	75.00	33.33	0.00–100.00
EORTC QLQ-PR25	Urinary symptoms	150	20.15 ± 15.35	16.67	19.05	0.00–100.00
	Incontinence aid	27	20.99 ± 33.52	0.00	33.33	0.00–100.00
	Bowel symptoms	89	5.71 ± 8.39	0.00	8.33	0.00–50.00
	Hormonal symptoms	130	12.68 ± 9.66	11.11	16.67	0.00–44.44
	Sexual activity	127	48.82 ± 25.73	50.00	33.33	0.00–100.00
	Sexual functioning	96	76.53 ± 18.85	83.33	25.00	0.00–100.00

*n*—number of available observations for the respective variable; M—mean; SD—standard deviation; Me—median; IQR—interquartile range; Min—minimum; Max—maximum; AIS—Acceptance of Illness Scale. Values of *n* < 150 indicate missing or not-applicable data for the respective variable.

**Table 2:** Postoperative characteristics of patients who completed the follow-up assessment (3–4 months after robotic radical prostatectomy), including disease acceptance (AIS), psychological adjustment to cancer (Mini-MAC), and quality of life according to the EORTC QLQ-C30 and EORTC QLQ-PR25 (N = 93).

Instrument	Variable	*n*	M ± SD	Me	IQR	Min–Max
AIS		93	35.08 ± 5.82	37.00	3.00	8.00–40.00
Mini-MAC	Anxious preoccupation	93	15.27 ± 3.62	15.00	4.00	7.00–27.00
	Fighting spirit	93	20.92 ± 3.12	21.00	4.00	12.00–28.00
	Helplessness–hopelessness	93	12.57 ± 3.33	13.00	3.00	7.00–27.00
	Positive re-evaluation	93	20.54 ± 2.74	20.00	4.00	14.00–26.00
	Constructive style	93	41.46 ± 4.58	42.00	5.00	29.00–51.00
	Destructive style	93	27.84 ± 6.55	28.00	8.00	14.00–53.00
EORTC QLQ-C30	Physical functioning	93	87.67 ± 17.91	93.33	20.00	6.67–100.00
	Role functioning	93	83.69 ± 19.81	83.33	33.33	16.67–100.00
	Emotional functioning	93	86.47 ± 22.08	100.00	16.67	8.33–100.00
	Cognitive functioning	93	93.73 ± 14.73	100.00	0.00	33.33–100.00
	Social functioning	93	86.74 ± 22.06	100.00	16.67	0.00–100.00
	Fatigue	93	16.49 ± 17.84	11.11	22.22	0.00–88.89
	Nausea and vomiting	93	5.20 ± 17.55	0.00	0.00	0.00–100.00
	Pain	93	16.85 ± 18.95	16.67	16.67	0.00–83.33
	Dyspnoea	92	8.33 ± 19.53	0.00	0.00	0.00–66.67
	Insomnia	93	9.32 ± 18.63	0.00	0.00	0.00–66.67
	Appetite loss	93	5.73 ± 14.43	0.00	0.00	0.00–66.67
	Constipation	93	18.28 ± 27.15	0.00	33.33	0.00–100.00
	Diarrhoea	93	11.47 ± 24.32	0.00	0.00	0.00–100.00
	Global health status/QoL	93	71.86 ± 17.93	75.00	16.67	16.67–100.00
EORTC QLQ-PR25	Urinary symptoms	93	15.62 ± 16.72	8.33	16.67	0.00–91.67
	Incontinence aid	38	40.35 ± 35.65	33.33	66.67	0.00–100.00
	Bowel symptoms	93	5.73 ± 13.46	0.00	8.33	0.00–58.33
	Hormonal symptoms	93	9.20 ± 11.19	5.56	16.67	0.00–50.00
	Sexual activity	93	24.91 ± 26.08	16.67	33.33	0.00–100.00
	Sexual functioning	54	60.80 ± 24.90	66.67	33.33	0.00–100.00

*n*—number of available observations for the respective variable; M—mean; SD—standard deviation; Me—median; IQR—interquartile range; Min—minimum; Max—maximum; AIS—Acceptance of Illness Scale. Values of *n* < 93 indicate missing or not-applicable data for the respective variable.

**Table 3:** Longitudinal comparison of illness acceptance (AIS), psychological adjustment to cancer (Mini-MAC), and quality of life (EORTC QLQ-C30 and EORTC QLQ-PR25) before and 3–4 months after robot-assisted radical prostatectomy.

Instrument	N Pairs	Before Surgery, Me (IQR)	After Surgery, Me (IQR)	Z	Unadjusted *p*	Holm-Adjusted *p*	*r*
AIS	92	34.00 (10.00)	37.00 (3.25)	2.066	0.039	0.465	0.22
Mini-MAC, anxious preoccupation	93	13.00 (6.00)	15.00 (4.00)	3.046	0.002	0.037	0.32
Mini-MAC, fighting spirit	93	24.00 (5.00)	21.00 (4.00)	−5.205	<0.001	<0.001	0.54
Mini-MAC, helplessness–hopelessness	92	9.00 (6.00)	13.00 (3.00)	4.338	<0.001	<0.001	0.45
Mini-MAC, positive re-evaluation	93	21.00 (4.00)	20.00 (4.00)	−1.399	0.162	1.000	0.15
Mini-MAC, constructive style, sten score	93	6.00 (2.00)	6.00 (1.00)	−4.295	<0.001	<0.001	0.45
Mini-MAC, destructive style, sten score	92	2.00 (2.00)	3.00 (2.00)	3.797	<0.001	0.003	0.40
EORTC QLQ-C30, physical functioning	93	86.67 (20.00)	93.33 (20.00)	2.645	0.008	0.123	0.27
EORTC QLQ-C30, role functioning	92	100.00 (20.83)	91.67 (33.33)	−1.064	0.287	1.000	0.11
EORTC QLQ-C30, emotional functioning	92	75.00 (18.06)	100.00 (16.67)	3.952	<0.001	0.001	0.41
EORTC QLQ-C30, cognitive functioning	92	83.33 (33.33)	100.00 (0.00)	4.933	<0.001	<0.001	0.51
EORTC QLQ-C30, social functioning	92	83.33 (33.33)	100.00 (16.67)	1.159	0.246	1.000	0.12
EORTC QLQ-C30, fatigue	93	11.11 (11.11)	11.11 (22.22)	−1.047	0.295	1.000	0.11
EORTC QLQ-C30, nausea and vomiting	92	0.00 (0.00)	0.00 (0.00)	0.423	0.673	1.000	0.04
EORTC QLQ-C30, pain	93	16.67 (33.33)	16.67 (16.67)	−1.409	0.159	1.000	0.15
EORTC QLQ-C30, dyspnoea	91	0.00 (0.00)	0.00 (0.00)	1.381	0.167	1.000	0.14
EORTC QLQ-C30, insomnia	92	0.00 (33.33)	0.00 (0.00)	−1.148	0.251	1.000	0.12
EORTC QLQ-C30, appetite loss	92	0.00 (33.33)	0.00 (0.00)	−2.364	0.018	0.253	0.25
EORTC QLQ-C30, constipation	92	33.33 (0.00)	0.00 (33.33)	−3.982	<0.001	0.001	0.42
EORTC QLQ-C30, diarrhoea	92	33.33 (33.33)	0.00 (0.00)	−3.405	<0.001	0.011	0.36
EORTC QLQ-C30, global health status/QoL	92	75.00 (33.33)	75.00 (16.67)	−1.220	0.223	1.000	0.13
EORTC QLQ-PR25, urinary symptoms	93	16.67 (20.83)	8.33 (16.67)	−2.064	0.039	0.465	0.21
EORTC QLQ-PR25, intestinal symptoms	54	0.00 (8.33)	0.00 (0.00)	−0.907	0.364	1.000	0.12
EORTC QLQ-PR25, hormonal symptoms	79	11.11 (16.67)	5.56 (16.67)	−2.171	0.030	0.389	0.24
EORTC QLQ-PR25, sexual activity	76	41.67 (33.33)	16.67 (37.50)	−4.602	<0.001	<0.001	0.53

Notes: N pairs = number of complete paired observations; Me = median; IQR = interquartile range. Z is the standardized, tie-adjusted Wilcoxon signed-rank statistic. Two-sided asymptotic unadjusted *p*-values were calculated from the corresponding Z statistics, without continuity correction. Zero pre-post differences were excluded from ranking, whereas tied non-zero absolute differences were assigned average ranks. Effect size *r* was calculated as the absolute value of Z divided by the square root of N, where N denotes all complete paired observations, including zero-difference pairs. Holm-adjusted *p*-values were calculated from the full-precision unadjusted *p*-values across the defined family of 25 comparisons. The conditional incontinence aid and sexual-functioning scores were analysed separately.

## Data Availability

The data presented in this study are available upon request from the corresponding author.

## Supplementary Materials

The following supporting information can be downloaded at: https://www.mdpi.com/article/10.3390/cancers18172774/s1. Supplementary Table S1: Baseline comparison of participants who completed postoperative follow−up and those lost to follow−up; Supplementary Table S2: Paired changes in incontinence−aid use among participants who completed both assessments (N = 93); Supplementary Table S3: Paired changes in the applicability of the EORTC QLQ−PR25 sexual−functioning domain among participants who completed both assessments (N = 93); Supplementary Table S4. Clinical and oncological characteristics of the study group (N = 150); Supplementary Table S5. Postoperative complications in the study group (N = 150).
