# Supplementary material for: Longitudinal Changes in Illness Acceptance, Psychological Adjustment, and Quality of Life After Robot-Assisted Radical Prostatectomy
Source: Cancers (Basel). 2026 Aug 26;18(17):2774. doi: 10.3390/cancers18172774 (PMC13565037; doi:10.3390/cancers18172774)
Supplement: Supplementary file 1 [file cancers-18-02774-s001.zip › cancers-4482421-supplementary.pdf]

Supplementary Table S1

Baseline comparison of participants who completed postoperative follow–up and those lost to follow–up

Instrument	Variable	Completers, n	Mean	Non-completers, n	Mean	SMD	Exploratory Mann–Whitney *p*
AIS	Acceptance of illness	92	33.01	57	31.44	0.21	0.224
Mini-MAC	Anxious preoccupation	93	13.38	57	14.28	−0.21	0.177
	Fighting spirit	93	23.44	57	23.46	−0.01	1.000
	Helplessness–hopelessness	92	10.38	57	11.19	−0.22	0.173
	Positive re-evaluation	93	21.13	57	21.70	−0.18	0.293
	Constructive style, sten score	93	6.67	57	6.86	−0.14	0.480
	Destructive style, sten score	92	2.13	57	2.44	−0.22	0.249
EORTC QLQ-C30	Physical functioning	93	81.77	57	83.74	−0.13	0.897
	Role functioning	92	86.96	57	88.60	−0.08	0.924
	Emotional functioning	92	75.94	57	75.29	0.04	0.635
	Cognitive functioning	92	81.34	57	85.96	−0.28	0.195
	Social functioning	92	84.06	57	84.21	−0.01	0.869
	Fatigue	93	18.40	57	19.49	−0.07	0.268
	Nausea and vomiting	92	3.26	57	1.75	0.19	0.195
	Pain	93	20.61	57	20.18	0.03	0.619
	Dyspnoea	92	5.44	57	7.02	−0.10	0.325
	Insomnia	92	13.04	57	13.45	−0.02	0.894
	Appetite loss	92	10.87	57	11.70	−0.05	0.691
	Constipation	92	33.33	57	32.16	0.05	0.783
	Diarrhoea	92	22.83	57	25.15	−0.11	0.471
	Global health status/QoL	92	74.46	57	73.54	0.04	0.710
EORTC QLQ-PR25	Urinary symptoms	93	19.50	57	21.23	−0.11	0.231
	Incontinence aid score	15	24.44	12	16.67	0.23	0.650
	Bowel symptoms	54	6.17	35	5.00	0.14	0.877
	Hormonal symptoms	79	12.26	51	13.32	−0.11	0.488
	Sexual activity	76	48.25	51	49.67	−0.06	0.662
	Sexual functioning	58	73.95	38	80.48	−0.35	0.227

Notes: AIS, Acceptance of Illness Scale; EORTC QLQ-C30, European Organisation for Research and Treatment of Cancer Quality of Life Questionnaire Core 30; EORTC QLQ-PR25, prostate cancer-specific quality-of-life module; Mini-MAC, Mini-Mental Adjustment to Cancer Scale; SMD, standardized mean difference; QoL, quality of life. Completers were participants who completed the postoperative assessment (n = 93), whereas non-completers were participants lost to follow-up (n = 57). Values are based on available baseline observations; therefore, outcome-specific sample sizes vary because of missing or not-applicable data. SMDs were calculated as the mean score among completers minus the mean score among non-completers, divided by the pooled

standard deviation. Consequently, negative SMD values indicate higher baseline scores among non-completers, whereas positive values indicate higher scores among completers. Mann–Whitney U tests were exploratory and were not included in the family of paired outcome comparisons subjected to Holm adjustment. The incontinence aid and sexual-functioning scores are conditional EORTC QLQ-PR25 outcomes and were available only for participants for whom the respective items were applicable.

Supplementary Table S2.

Paired changes in incontinence−aid use among participants who completed both assessments (N = 93).

Incontinence−aid use before surgery	No use at follow−up *n*	Use at follow−up *n*	Total *n*
No use before surgery	47	31	78
Use before surgery	8	7	15
Total	55	38	93

The paired change in incontinence−aid use was evaluated using an exact two−sided McNemar test ($p < 0.001$). Percentages based on all 93 follow−up completers were 16.1% before surgery and 40.9% at follow−up.

Supplementary Table S3.

Paired changes in the applicability of the EORTC QLQ−PR25 sexual−functioning domain among participants who completed both assessments (N = 93)

Applicability before surgery	Not applicable at follow−up, *n*	Applicable at follow−up, *n*	Total, *n*
Not applicable before surgery	13	22	35
Applicable before surgery	26	32	58
Total	39	54	93

The sexual−functioning domain of the EORTC QLQ−PR25 is conditional and was scored only for participants for whom the corresponding items were applicable. Of the 93 participants who completed both assessments, the domain was applicable to 58 participants (62.4%) before surgery and 54 participants (58.1%) at follow−up. Twenty−two participants entered the subgroup for whom the domain was applicable, whereas 26 participants left this subgroup; the domain remained applicable at both assessments for 32 participants. The paired change in domain applicability was evaluated using an exact two−sided McNemar test and was not statistically significant ($p = 0.665$). This analysis concerns domain applicability and should not be interpreted as a direct comparison of sexual-functioning scores.

Supplementary Table S4. Clinical and oncological characteristics of the study group (N = 150).

Clinical and oncological variable	Mean ± SD	Median (IQR)	Minimum	Maximum
PSA (ng/mL)	9.51 ± 5.98	7.70 (5.27)	2.20	33.00
PI-RADS score	4.12 ± 0.88	4.00 (1.00)	1.00	5.00
Gleason score	6.70 ± 0.95	7.00 (1.00)	6.00	10.00
ISUP grade	2.11 ± 1.14	2.00 (2.00)	1.00	5.00
Briganti score	0.11 ± 0.20	0.04 (0.09)	0.01	2.00

Abbreviations: PSA, prostate-specific antigen; PI-RADS, Prostate Imaging Reporting and Data System; ISUP, International Society of Urological Pathology; SD, standard deviation; IQR, interquartile range.

Low risk of lymph node metastases was defined as a Briganti score <5%. Overall, 77 patients (51.3%) had a Briganti score <5% and were classified as low risk, whereas 73 patients (48.7%) had a Briganti score ≥5%.

Supplementary Table S5. Postoperative complications in the study group (N = 150)

Postoperative complications	n	%
No	137	91.3
Yes	13	8.7

Abbreviations: n, number of patients.
